# The Effects of Exercise Difficulty and Time-of-Day on the Perception of the Task and Soccer Performance in Child Soccer Players

**DOI:** 10.3390/children8090793

**Published:** 2021-09-10

**Authors:** Liwa Masmoudi, Adnene Gharbi, Cyrine H’Mida, Khaled Trabelsi, Omar Boukhris, Hamdi Chtourou, Mohamed Amine Bouzid, Cain C. T. Clark, Nizar Souissi, Thomas Rosemann, Beat Knechtle

**Affiliations:** 1 Institut Supérieur du Sport et de l’éducation Physique de Sfax, Université de Sfax, Sfax 3000, Tunisia; liwa.masmoudi@yahoo.fr (L.M.); adnenegharbi@yahoo.fr (A.G.); sirinehmida7@gmail.com (C.H.); trabelsikhaled@gmail.com (K.T.); omarboukhris24@yahoo.com (O.B.); h_chtourou@yahoo.fr (H.C.); mohamedamine.bouzid@isseps.usf.tn (M.A.B.); n_souissi@yahoo.fr (N.S.); 2 Research Laboratory: Education, Motricité, Sport et Santé, EM2S, LR19JS01, High Institute of Sport and Physical Education of Sfax, University of Sfax, Sfax 3000, Tunisia; 3 Activité Physique, Sport et Santé, UR18JS01, Observatoire National du Sport, Tunis 1003, Tunisia; 4 Centre for Intelligent Healthcare, Coventry University, Coventry CV1 5FB, UK; ad0183@coventry.ac.uk; 5 Institute of Primary Care, University of Zurich, 8006 Zurich, Switzerland; thomas.rosemann@usz.ch; 6 Medbase St. Gallen Am Vadianplatz, 9000 St. Gallen, Switzerland

**Keywords:** perceived difficulty, psychomotor performance, diurnal variation, child, mood, soccer

## Abstract

In soccer, accurate kicking skills are important determinants of successful performance. A successful kick must meet several criteria, including speed, accuracy, and timing. In fact, players who are able to kick the ball more accurately under various difficulties, such as time pressure, space constraints, the opponent’s pressure, and the distance between the kicking point and the goal, have a clear advantage during soccer games. The aim of the present study was to investigate the effect of exercise difficulty and time-of-day on perceived task difficulty and kicking performance. Accordingly, 32 boys (age: 11 ± 0.7 years; height: 1.45 ± 0.07 m; body-mass: 38.9 ± 7.8 kg) performed shooting accuracy tests under two difficulty levels (distance (long-distance (LD) vs. short-distance (SD)) and time pressure (Without-time-pressure (WTP) vs. With-time-pressure (TP)) at 08:00 h and 17:00 h. Absolute-error, variable-error, and constant-error were evaluated during the kicking tasks, in addition to ball velocity and shooting quality. Moreover, rating-of-perceived-exertion score (RPE), feeling-scale (FS), and perceived difficulty were completed immediately at the end of each test. The results showed that shooting quality was not affected by the time-of-day, but it was better in WTP vs. TP (*p* < 0.05), and in SD vs. LD (*p* < 0.05), respectively. Higher values for FS and lower values for RPE were observed in the morning compared to the afternoon (*p* < 0.05) and in WTP vs. TP (*p* < 0.05). In conclusion, specific soccer skills of boys were not time-of-day dependent, but they may be associated with time pressure and task difficulty.

## 1. Introduction

The influence of time-of-day effects on psycho-physiological, cognitive, and physical performance assessments has been widely studied [1]. Indeed, in children, previous studies have reported that muscle power [2], strength [3], agility [4], and aerobic fitness [2,5,6] were better at the end of the afternoon compared to the morning hours. Likewise, some psycho-physiological functions, such as cardiovascular and metabolic responses to exercise [7], mental [6], psychomotor [8], and cognitive performances [9,10,11] have been found to be better in the late afternoon, approximately corresponding to the circadian peak of body temperature [4,12]. However, contrastingly, Huguet et al. [13] reported that several simple coordination skills tend to peak earlier in the day compared to complex coordinative skills [13]. Discrepancies between the findings reported in the literature could be related to differences in the mental load of the task, the type of the task, and the perceived difficulty.

In this context, Elghoul et al. [9,10] investigated the time-of-day effects on dart-throwing performance from short and long distance and the perception of the difficulty of the task. Accordingly, the authors reported better performance in both long and short distance at 17:00 h compared to 07:00 h, concomitant to significant reductions in perceived task difficulty from 07:00 h to 17:00 h.

Pertaining to the previous studies, a number of limitations may be manifest related to the chosen task and method employed. Moreover, the number of participants for these studies was small, precluding any firm conclusions. Furthermore, assessment of accuracy was performed across a number of points using different protocols (i.e., scores per trial, numbers of zeros scored, radial error); indeed, this method of accuracy estimation is inadequate and presents low reliability [14]. It has been posited that the direct measures, such as the absolute error, variable error, and constant error, as well as velocity (the kicked object) could provide both a more valid and more informative description of the kicking performance [14].

Goal scoring is an important element in soccer [15] that necessitates, particularly during key moments of the match-play, high velocity and accuracy of shooting. Indeed, previous studies have reported a direct relationship between speed of leg movement and subsequent ball speed [16]. However, it should be acknowledged that an increased execution speed can deleteriously affect accuracy [17]. In goal-directed aiming tasks, increased time pressure and increased target distance will impact the index of difficulty [18]. Therefore, shooting at a target under time-pressured conditions, in response to the demanding nature of competitive soccer, would conceivably elicit an increased execution speed and, in turn, a decreased accuracy. Indeed, previous studies addressing instep kicks have proven that, by prioritizing accuracy, the ball kicking velocity can be significantly reduced [19]. Moreover, in children, Frikha et al. [20] found that the instep kicking accuracy and missed kicks were significantly decreased in time-pressured vs. free conditions. In addition, the perception of difficulty was higher in time-pressured vs. free conditions [20].

A dearth of studies has investigated the effects of time-of-day on coordination skills in soccer [4,12]. Of the available data, some studies reported that kicking accuracy, juggling performance, ball control with the body, and ball control with the head were not affected by the time-of-day [4,12]. However, other investigations showed that coordination skills of kicking accuracy, juggling, and dribbling performance were better in the afternoon compared to the morning [6,21].

Indeed, little is known about the association of circadian rhythms and level of difficulty. Moreover, the diurnal variation of coordination skill performance in children remains scarcely investigated. Therefore, we hypothesized that kicking accuracy would be better without time pressure (WTP) compared to time-pressure (TP) and for the short compared to the long distance. In addition, we hypothesized that psychomotor performance would be better in the afternoon compared to the morning hours. Accordingly, the aim of this study was to examine the effect of time-of-day (08:00 h and 17:00 h) and the level of difficultly (kicking WTP and kicking TP conditions from a long (10 m) and a short (6 m) distance) on kicking accuracy and perceived difficulty in child soccer players.

## 2. Materials and Methods

### 2.1. Participants

Thirty-two male soccer players (age: 11 ± 0.7 years; height: 1.45 ± 0.07 m [WHO Z-score = 0.03]; body-mass: 38.9 ± 7.8 kg [WHO Z-score = 0.75]; body mass index: 18.5 kg/m^2^ [WHO Z-score = 0.74]), from three teams of the first division Tunisian youth league, voluntarily participated in the study. They participated in four training sessions per week and one match at the weekend (usually on Sunday). All boys were classified as prepubertal (stage 1) by a pediatrician, according to Tanner criteria [22]. Participants were included in the present study if: (a) his age was between 10 and 12 years, (b) he had no history of neurological, musculoskeletal, or orthopedic disorders or had no history of lower extremity surgery or injury in the 6 months before testing that might have affected their physical ability, (c) he was able to understand and follow instructions, and (d) he had no cognitive or visual problems.

Before participation to the study, boys and their parents/guardians provided written informed consent, and they were informed that they could withdraw from the study at any time.

### 2.2. Procedure

After two familiarization sessions, boys performed a shooting accuracy tests (10 kicks) on a target of 1.2 m radius, under two difficulty levels (distance and time pressure). First, they performed the test from two distances: 6 m and 10 m. The distances of 6 m and 10 m were chosen arbitrarily to reflect short and long distances, respectively [23]. Second, to increase the difficulty level of the task for each distance, boys were instructed to complete the 10 kicks as accurately as possible, WTP and TP. During TP, the boys were asked to perform the test shot with a frequency of 1 shot every 3 s and during WTP they were asked to perform the test shot with a frequency of 1 shot every 6 s. The frequency of the shots was controlled by an audible signal emitted by an audio source.

**Figure 1:**
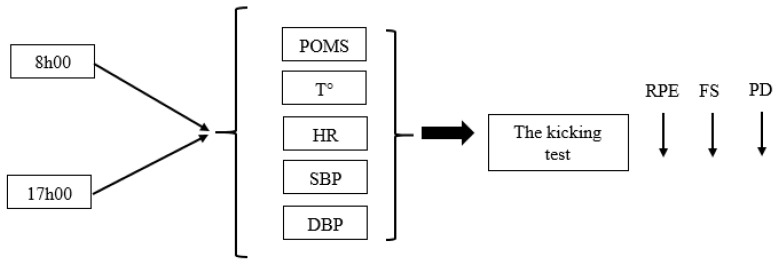
Schematic representation of the experimental design. POMS: Profile of mood states; T°: temperature; HR: heart rate; SBP: systolic blood pressure; DBP: diastolic blood pressure; RPE: rating of perceived exertion; FS: feeling scale; PD: perceived task difficulty.

The measurements were taken, in a randomized order, at two different time-of-day: 08:00 h and 17:00 h. For each test session, boys woke up at 06h30 and ate a standardized breakfast. At 12h30, boys ate a standardized iso-caloric lunch. From 12h30 onward, boys were allowed to consume only drinking water. At the beginning of each test session, boys were asked to complete the Profile of Mood States (POMS) questionnaire. In addition, intra-aural temperature (ThermoScan IRT 4520, Braun GmbH, Kronberg, Germany), heart rate (Polar Team System, Polar Electro Oy, Kempele, Finland), and systolic (SBP) and diastolic (DBP) blood pressure (Beurer, B.M 20, 89077 Ulm, Germany) were measured at the beginning of each session. Moreover, the boys indicated their rating of perceived exertion (RPE), feeling scale (FS), and perceived task difficulty (PD), immediately at the end of the kicking test (Figure 1).

### 2.3. Kicking Accuracy

**Figure 2:**
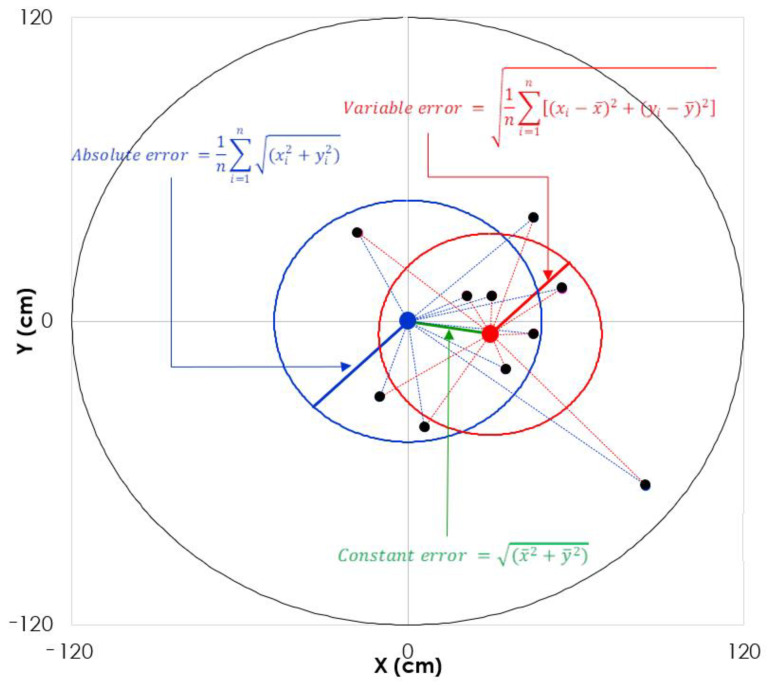
Schematic representation of the ten bullet impact points with the target and the three accuracy errors. The large black circle represents the target (1.2 m radius); The blue point represents the center of the target; The ten black dots represent the points of impact of the ball with the target; Blue dashed lines represent absolute errors; The radius of the blue circle represents the mean absolute error of the 10 shots; The red point represents the barycenter of the 10 black points; Red dashed lines represent relative errors; The radius of the red circle represents the average relative error of the 10 shots; The green line represents the constant error.

The kicking accuracy test consisted of performing 10 kicks from a point placed at 6 and 10 m from the target, respectively [23]. To determine the accuracy of the kick, we analyzed video recordings of each trial. Absolute error (AE), variable error (VE), and constant error (CE) were evaluated as the variables of kicking accuracy (Figure 2). Further, ball velocity (BV; measured via radar gun), and shooting quality (SQ; shooting accuracy divided by the time elapsed from hitting the ball to the bulls-eye) were determined.

### 2.4. Perceived Exertion Scale Rating

The RPE measure was obtained based on the Borg’s category ratio-scale (CR-10), as modified by Foster et al. [24]. Boys rated how hard the exercise felt from a scale of 0 (nothing at all) to 10 (maximal). The RPE scale is a reliable indicator of physical discomfort, has sound psychometric properties, and is strongly correlated with several other physiological measures of exertion [24].

### 2.5. Feeling Scale

Affect was measured using a FS [25]. The FS items were rated on an 11-point scale: +5, very good; +3, good; +1, fairly good; 0, neutral; −1, fairly bad; −3, bad; −5, very bad. The scale was used to measure the affective component of exercise, i.e., whether the exercise felt pleasant or unpleasant.

### 2.6. Perceived Difficulty

PD was assessed according to the difficulty perception −15 scale [26], a 15-points category scale, with 7 labels, from “extremely easy” to “extremely difficult”, symmetrically placed around a central label, “somewhat difficult”.

### 2.7. The Profile of Mood States (POMS)

The POMS is a self-report questionnaire, developed by McNair [27], including 65 items, measuring five negative moods (tension-anxiety, depression, anger-hostility, vigor-activity, fatigue, and confusion-bewilderment), one positive mood (vigor), and interpersonal relationships, and a total mood disturbance score (TMD). Five-point Likert scales, ranging from 0 (“not at all”) to 4 (“extreme”) were used.

TMD = (Tension + Depression + Anger + Fatigue + Confusion) − Vigor

### 2.8. Statistical Analysis

All statistical tests were processed using STATISTICA software (StatSoft, version 12, Paris, France). All values were expressed as mean ± SD. G*power software (version 3.1.9.2; Kiel University, Kiel, Germany) [28] was used to calculate the required sample size. Values for α were set at 0.05 and power at 0.8. Based on the study of Masmoudi et al. [12] and consensus between the authors, the effect size was estimated to be 0.49. Accordingly, the required sample size for this study was 28. Following normality confirmation using the Kolmogorov-Smirnov test, the data of the kicking accuracy variables (AE, VE, CE, BV, SQ), FS, RPE, and PD were analyzed using a three-way repeated measure analysis of variance (ANOVA) (2 conditions (WTP and TP) × 2 distances (SD and LD) × 2 time-of-day (08:00 h and 17:00 h), and, when appropriate, Bonferroni post-hoc tests were used to determine where significant differences existed. In addition, partial eta-squared (η_p_^2^) was calculated for these variables. Finally, differences in temperature, HR, SBP, DBP, and POMS were compared using paired-sample *t*-tests. Statistical significance was set, a priori, at *p* < 0.05.

## 3. Results

### 3.1. Psychomotor Parameters

#### 3.1.1. Kicking Accuracy

The 3-way ANOVA on AE indicated significant main effects of condition (F_(1,31)_ = 103.48; *p* < 0.001, η_p_^2^ = 0.769) and distance (F_(1,31)_ = 276.72; *p* < 0.001]; η_p_^2^ = 0.899); no significant main effect for time-of-day or significant interactions were found.

The Bonferroni post hoc test revealed that AE with WTP and TP was significantly lower in SD compared to LD (*p* < 0.001), and AE during SD and LD was higher for TP compared to WTP (*p* < 0.001) (Table 1).

The variable error revealed the same main effects for condition (F_(1,31)_ = 8.63; *p* < 0.006, η_p_^2^ = 0.218) and distance (F_(1,31)_ = 121.02; *p* < 0.001; η_p_^2^ = 0.796), without a significant main effect for time-of-day or significant interactions between these variables.

The Bonferroni post hoc test revealed that the variable error with WTP and TP was significantly lower in SD compared to LD (*p* < 0.001) (Table 1).

The CE revealed only a significant main effect for distance (F_(1,31)_ = 21.91; *p* < 0.001, η_p_^2^ = 0.414).

The Bonferroni post hoc test revealed that CE, with TP at only 17h00, was significantly lower in SD compared to LD (*p* = 0.007) (Table 1).

#### 3.1.2. Kicking Velocity

The 3-way ANOVA on ball velocity indicated significant main effects of condition (F_(1,31)_ = 61.26; *p* < 0.001, η_p_^2^ = 0.664) and distance: F_(1,31)_ = 21.11; *p* < 0.001; η_p_^2^ = 0.405); but no main significant effect for time-of-day or significant interactions were found.

The Bonferroni post hoc test revealed that the ball velocity with WTP was significantly lower in SD compared to LD (*p* < 0.05) (Table 1).

For shooting quality, we found significant main effects for condition (F_(1,31)_ = 101.25; *p* < 0.001, η_p_^2^ = 0.766) and distance (F_(1,31)_ = 532.6; *p* < 0.001; η_p_^2^ = 0.945). In addition, the results showed a significant condition × distance interaction (F_(1,31)_ = 6.25; *p* < 0.05, η_p_^2^ = 0.168). The Bonferroni post hoc testing revealed that shooting quality with SD was significantly better in WTP compared to TP (*p* < 0.001), and that shooting quality during TP was better for SD compared to LD (*p* < 0.001) (Table 1).

### 3.2. Perceived Difficulty and Psychological Parameters

For FS, the results showed significant main effects for time-of-day (F_(1,31)_ = 10.41; *p* = 0.003, η_p_^2^ = 0.251) and distance (F_(1,31)_ = 8.03; *p* < 0.01; η_p_^2^ = 0.206). In addition, significant interactions time-of-day × condition (F_(1,31)_ = 33.49; *p* < 0.001, η_p_^2^ = 0.519) and time-of-day × distance (F_(1,31)_ = 12.31; *p* < 0.001, η_p_^2^ = 0.284) were observed. The Bonferroni post hoc testing revealed that FS score was higher in TP at 08:00 h than 17:00 h (*p* < 0.001) and that FS score in TP was higher than WTP at 08:00 h (*p* < 0.001) and at 17:00 h (*p* < 0.01), respectively (Table 1). The FS score for the LD condition at 17:00 h was significantly lower than at 08:00 h (*p* < 0.001) and was lower during LD compared to SD in the two time-of-day (*p* < 0.001) (Table 1).

The PD was not affected by the time-of-day and time pressure. However, a significant main effect for distance (F_(1,31)_ = 34.67; *p* < 0.001, η_p_^2^ = 0.528) was observed. PD score was higher in LD compared to SD (*p* < 0.001). POMS subscale, TMD scores, and *t*-test results at the two measurements are presented in Table 2. The depression and the vigor scores were significantly higher at 17:00 h compared to 08:00 h (*p* < 0.05). However, anger, confusion, fatigue, inter-relation, and the TMD scores were not significantly affected by the time-of-day (Table 2).

Concerning RPE, our results showed a significant main effects for time-of-day (F_(1,31)_ = 21.41; *p* < 0.001; η_p_^2^ = 0.408), condition (F_(1,31)_ = 18.46; *p* < 0.001; η_p_^2^ = 0.373), and distance (F_(1,31)_ = 14.51; *p* < 0.001; η_p_^2^ = 0.319). The statistical analysis revealed higher RPE scores (i) at 17:00 h than 08:00 h (Table 1) (*p* < 0.05) and (ii) during LD and TP compared to SD and WTP, respectively (*p* < 0.05 and *p* < 0.05) (Table 2).

### 3.3. Physiological Parameters

Statistical analysis showed that intra-aural temperature and HR values were significantly higher at 17:00 h than 08:00 h (+ 3.4%, *p* < 0.001 and + 4.7%, *p* < 0.05, respectively, Table 3). However, no significant time-of-day effect was found for systolic and diastolic blood pressures (Table 3).

## 4. Discussion

The principal findings of the current study were (i) there was no significant time-of-day differences between 08:00 h and 17:00 h regarding kicking accuracy, (ii) kicking accuracy was higher without vs. with time pressure, (iii) feelings scale scores were greater in the morning vs. afternoon, and during SD vs. LD at 17h00, respectively, (iv) the RPE scores were higher during TP vs. WTP, and in the afternoon vs. the morning, respectively, and (v) the RPE scores were higher during LD vs. SD in TP, and TP vs. WTP in the LD condition, respectively.

The current study suggested that specific soccer skills of boys are not dependent on time-of-day. In line with the present study, Gharbi et al. [4] noted that soccer skills of boys did not differ between 07:00 h and 17:00 h. Indeed, the authors reported that, in Tunisian boys (mean age: 12.7 years), kicking accuracy and juggling performance remained unaffected by time-of-day. Similarly, Masmoudi et al. [12], in Tunisian children (mean age: 14.6 years), concluded that shooting accuracy was not impacted by time-of-day. However, some other soccer specific skills have been reported to be time-of-day dependent [4,12]. In this context, Gharbi et al. [4] showed that agility and dribbling were better in the afternoon vs. the morning; whilst Masmoudi et al. [12] concurred with this finding and reported that dribbling performance was better at 13:00 h and 17:00 h vs. 08:00 h. In this context, it has been reported that the diurnal rhythm of intra-aural temperature could explain the diurnal variation of dribbling performance [12]. In fact, the increase in intra-aural temperature in the afternoon could enhance the conduction velocity of the action potentials as well as metabolic reactions, improving muscle contraction during the dribbling test. However, although intra-aural temperature values were significantly higher at 17:00 h than 08:00 h, there were no significant time-of-day differences between 08:00 h and 17:00 h for the kicking accuracy. Therefore, the present study indicates that technical specific skills (i.e., kicking accuracy) is not associated with the diurnal rhythm of intra-aural temperature. It is difficult to explain these discrepancies between studies pertaining to diurnal variation of soccer specific skills in children; however, it is possible that dribbling performance is related, at least in part, to the sprint and agility qualities that have been shown to be better in the afternoon [4,12]. Accordingly, Bernard et al. [29] reported that sprint performance was time-of-day dependent, with better outcomes in the afternoon compared to the morning. Similarly, other short-term maximal performances (e.g., vertical jump, repeated sprints, etc.) have been shown to be better in the afternoon vs. morning [2,3,5,30]. It is conceivable, as shown in the present results, that technical specific skills (i.e., kicking accuracy) of soccer players are not affected by the time-of-day of testing; however, when technical skills are coupled with physical abilities (e.g., agility, dribbling, etc.), significant diurnal variation of children’ performances may be observed.

The lack of a significant time-of-day effect on kicking accuracy could be, at least partly, related to the typical time-of-day of training (i.e., soccer training sessions in the academy that took place in the morning hours). Indeed, in boys, Souissi et al. [3] noted that regular training in the morning hours could significantly affect the diurnal variation of short-term maximal performance. Given that boys recruited for the present study regularly trained in the morning hours, we could speculate that this training adaptation could, partially, explain the non-significant time-of-day effect on kicking accuracy. Likewise, better feelings scores in the morning (i.e., the reported time-of-day of worse performance) were observed, which could indicate that boys prefer performing tests in the morning hours, as they regularly trained at this time-of-day, and this could improve their performance and affect the normal diurnal variation of performance. This suggests that a lower psychological discomfort reported in the morning could have contributed to the better performance reported in that time-of-day. In support of this idea, the TMD scores were not significantly affected by the time-of-day, which could explain the lack of a significant time-of-day effect on kicking accuracy. In this context, we also showed that RPE scores were higher in the afternoon hours (indicating higher physiological discomfort in the afternoon vs. morning), which could suggest that boys in the present study were better prepared to perform the tests in the morning hours. In addition, the higher motivation of participants in the morning could explain also the previously reported morning to afternoon difference. Likewise, HR values were higher at 17:00 h than 08:00 h. The higher values of RPE and HR in the afternoon hours could be related to the amount of activity undertaken in the afternoon by children. Additionally, the PD was not affected by the time-of-day, which could also explain the lack of a significant time-of-day effect on kicking accuracy. Indeed, PD mainly reflects the amount of resources, or effort, that boys have devoted to the task in order to reach a given level of performance [9]. In fact, it has been shown that, in children, fatigue during testing of soccer-specific skills is not affected by the time-of-day [12]. Furthermore, SBP and DBP did not vary with time-of-day, which could explain why specific soccer skills of boys were not time-of-day dependent.

For task difficulty, the present study showed that performance was better WTP in comparison to TP. To the best of our knowledge, this is the first study examining the effect of time-pressure on kicking accuracy in boys according to different times-of-day. During a dart-throwing task, Elghoul et al. [9,10] investigated the task difficulty at 07:00 h and 17:00 h, and reported that the PD was higher with LD vs. SD due to geometry. However, in the present study, we did not report a significant time-of-day effect on PD. The discrepancies between the findings of the present study and those of Elghoul et al. [9,10] could be related to the utilized psychomotor task (kicking accuracy (coordination visual perception and lower body) vs. dart-throwing (coordination visual perception and upper body)). The effect of PD on psychomotor performance should be interpreted with caution because PD was measured immediately following the kicking accuracy test; thus, due to the timing of the measurement, test performance may be influenced by the players’ perception of their own performance.

## 5. Strengths and Weaknesses

The present study is the first to investigate the effect of time-pressure on kicking accuracy, in boys, according to different times-of-day. A limitation of the current study was that dietary intake analysis was not realized before the study; however, as indicated, standardized breakfast and iso-caloric lunch were given to all boys. Another limitation of the present study was the lack of recording rectal temperature; only oral temperature was measured for ethical reasons. Furthermore, all participants were male soccer players, so our results do not generalize all populations. Finally, although we conducted an *a priori* power calculation to discern suitable statistical power, the presence of multiplicity in the statistical analyses (i.e., ANOVA) may have impacted on subsequent error rates. Nevertheless, we elected to utilize Bonferroni corrected post-hoc test, which is a conservative method of correcting for multiple testing. Notwithstanding the efforts made to ensure reliable results, the authors advocate for future studies that consider the time-of-day impact, across multiple days and bouts, to provide a more detailed understanding into the potential variability and error related to these findings.

## 6. Conclusions

Kicking accuracy of boys is better without time-pressure vs. with time-pressure. However, there was no-significant time-of-day effect on kicking accuracy or in perceived difficulty. Further studies are needed to explain the findings of the present study. From a practical point of view, sports scientists, clinicians, and coaches should consider the effect of time-of-day in studies, training programs, and competitive events involving boys’ kicking accuracy.

## Figures and Tables

**Table 1:** Kicking accuracy and velocity, perceived difficulty, feeling score, and rating of perceived exertion (RPE) recorded at 08:00 h and 17:00 h during the short (SD) and long (LD) distances for the two experimental conditions (without time pressure (WTP) and with time pressure (TP)).

Variable		WTP		TP	
		SD	LD	SD	LD
**Absolute error (cm)**	08:00 h	53.5 ± 12.7	79.1 ± 12.5 ^1^	67.9 ± 13.6 ^2^	93.3 ± 10.3 ^1,2^
	17:00 h	53.8 ± 13.2	76.8 ± 14.3 ^1^	65.9 ± 16.8 ^2^	91.8 ± 10.5 ^1,2^
**Variable error (cm)**	08:00 h	47.9 ± 12	67.1 ± 13.7 ^1^	50.1 ± 13.1	71.9 ± 13.3 ^1^
	17:00 h	50.7 ± 13.7	67.7 ± 14.4 ^1^	52.1 ± 17.2	72.4 ± 11.3 ^1^
**Constant error (cm)**	08:00 h	19.6 ± 14.8	27.7 ± 16.4	22.5 ± 16.1	29.1 ± 15.5
	17:00 h	18.7 ± 12.6	28.7 ± 14.9	21 ± 9.1	32 ± 17.3 ^1^
**Ball velocity (m/s)**	08:00 h	9.6 ± 1.3	10.5 ± 1.7 ^1^	8.7 ± 1.2	9.1 ± 1.4
	17:00 h	9.6 ± 1.4	10.5 ± 1.6 ^1^	8.7 ± 1.7	9.2 ± 1.3
**Shooting quality (m/s)**	08:00 h	1.06 ± 0.25	0.43 ± 0.14 ^1^	0,75 ± 0.22 ^2^	0.24 ± 0.1 ^1,2^
	17:00 h	0.85 ± 0.22	0.8 ± 0.17 ^1^	0.96 ± 0.29 ^2^	0.84 ± 0.14 ^1,2^
**Perceived difficulty (A.U)**	08:00 h	4.84 ± 2.33	6.31 ± 1.97 ^1^	5.03 ± 2.61	5.81 ± 2.31 ^1^
	17:00 h	5.56 ± 2.68	6.47 ± 2.63	4.75 ± 2.37	7.03 ± 2.75 ^1^
**Feeling score (A.U)**	08:00 h	2.19 ± 2.62	2.28 ± 1.53	3.16 ± 1.92 ^2^	3.34 ± 2.22 ^2^
	17:00 h	3.19 ± 1.8 ^3^	2.09 ± 1.49 ^1^	2.69 ± 1.71	0.63 ± 1.68 ^1,2,3^
**RPE (A.U)**	08:00 h	1.0 ± 1.02	1.16 ± 0.88 ^1^	0.78 ± 1.16	1.63 ± 1.62 ^1^
	17:00 h	1.03 ± 1.18	1.34 ± 0.6	1.5 ± 1.34 ^3^	2.16 ± 1.14 ^2,3^

^1^: Significantly different compared to SD at *p* < 0.05; ^2^: Significantly different compared to WTP at *p* < 0.05; ^3^: Significantly different compared to 08:00 h at *p* < 0.05.

**Table 2:** The POMS subscale and TMD scores recorded at the two times of day.

Variable	08:00 h	17:00 h	*t*-Test	*p*-Value
**Anxiety (A.U)**	2.6 ± 3.1	3.2 ± 3.7	0.69	0.496
**Anger (A.U)**	5.5 ± 4	5 ± 5.2	0.43	0.669
**Confusion (A.U)**	2.1 ± 3.7	2 ± 2.2	0.12	0.905
**Depression (A.U)**	1.6 ± 2.1	3.1 ± 4.2	2.47	0.019
**Fatigue (A.U)**	2.3 ± 2.7	2.3 ± 3.2	0.06	0.955
**Vigor (A.U)**	26 ± 4.3	28.1 ± 4.2	2.19	0.036
**Interpersonal-Relationship (A.U)**	23.1 ± 5	23.3 ± 4.4	0.12	0.908
**TMD (A.U)**	−12.1 ± 11.8	−12.5 ± 18.2	0.13	0.896

**Table 3:** Intra-oral temperature, heart rate (HR), systolic blood pressure (SBP) and diastolic blood pressure (DBP) recorded at the two time-of-day.

Variable	08:00 h	17:00 h	*t*-Test	*p*-Value
**Temperature (°C)**	35 ± 0.8	36.2 ± 0.5	7.46	<0.001
**HR (b·min^−1^)**	84 ± 9	88 ± 11	2.26	0.031
**SBP (mmHg)**	11.4 ± 0.7	11.5 ± 1.1	0.21	0.833
**DBP (mmHg)**	6.5 ± 0.7	6.6 ± 1.2	0.33	0.741

## Data Availability

Data available on request due to privacy or ethical restrictions.
